# Characterization of a unique technique for culturing primary adult human epithelial progenitor/“stem cells”

**DOI:** 10.1186/1471-5945-12-8

**Published:** 2012-06-24

**Authors:** Cynthia Luz Marcelo, Antonio Peramo, Amala Ambati, Stephen E Feinberg

**Affiliations:** 1 Department of Surgery, Section of Plastic and Reconstructive Surgery, University of Michigan, MSRBII, A560 1150 W. Medical Center Dr., Ann Arbor, MI, 48109, USA; 2 Oral and Maxillofacial Surgery, School of Dentistry, MSRBII, A560 1150 W. Medical Center Dr., Ann Arbor, MI, 48109, USA; 3 Department of Surgery, Oral and Maxillofacial Surgery, Section of Oral and Maxillofacial Surgery, School of Dentistry, University of Michigan, MSRBII, A560 1150 W. Medical Center Dr., Ann Arbor, MI, 48109, USA

## Abstract

**Background:**

Primary keratinocytes derived from epidermis, oral mucosa, and urothelium are used in construction of cell based wound healing devices and in regenerative medicine. This study presents *in vitro* technology that rapidly expands keratinocytes in culture by growing monolayers under large volumes of serum-free, essential fatty acid free, low calcium medium that is replaced every 24 hrs.

**Methods:**

Primary cell cultures were produced from epidermal skin, oral mucosa and ureter by trypsinization of tissue. Cells were grown using Epilife medium with growth factors under high medium volumes. Once densely confluent, the keratinocyte monolayer produced cells in suspension in the overlying medium that can be harvested every 24 hrs. over a 7–10 day period. The cell suspension (approximately 8 X 10^5^ cells/ml) is poured into a new flask to form another confluent monolayer over 2–4 days. This new culture, in turn produced additional cell suspensions that when serially passed expand the cell strain over 2–3 months, without the use of enzymes to split the cultures. The cell suspension, called **e**pithelial **P**op **U**p **K**eratinocytes (ePUKs) were analyzed for culture expansion, cell size and glucose utilization, attachment to carrier beads, micro-spheroid formation, induction of keratinocyte differentiation, and characterized by immunohistochemistry.

**Results:**

The ePUKs expanded greatly in culture, attached to carrier beads, did not form micro-spheroids, used approximately 50% of medium glucose over 24 hrs., contained a greater portion of smaller diameter cells (8–10 microns), reverted to classical appearing cultures when returned to routine feeding schedules (48 hrs. and 15 ml/T-75 flask) and can be differentiated by either adding 1.2 mM medium calcium, or essential fatty acids. The ePUK cells are identified as cycling (Ki67 expressing) basal cells (p63, K14 expressing).

**Conclusions:**

Using this primary culture technique, large quantities of epithelial cells can be generated without the use of the enzyme trypsin to split the cultures. The cells are small in diameter and have basal cell progenitor/”stem” (P/SC) cell characteristics induced by daily feeding with larger than normal medium volumes. The ePUK epithelial cells have the potential to be used in regenerative medicine and for basic studies of epithelia P/SC phenotype.

## Background

A multitude of methods are being developed to obtain non-transformed adaptable cells, i.e. cells with functional plasticity, to construct artificial tissue for transplant, to “correct” specific systemic diseases and as a source for cell-mediated wound healing therapies [1-5]. The establishment of a technique to grow adult somatic cells with maximum plasticity, from human tissue, can circumvent many of the well-known and currently debated ethical and scientific problems associated with the use of ESCs (embryonic stem cells) and iPSCs (induced pluripotent stem cells) [6]. In an attempt to eliminate the problems associated with retroviral transduction an approach to generate adult stem cells using small molecules to epigenetically reprogram the cells (transdifferentiation) has been advocated [7,8]. The novel method described in our report is an *in vitro* culturing technique for isolating and maintaining adult human epithelial primary cells in culture that allows for excellent growth of small diameter cells expressing characteristic epithelial cell markers. The primary cells grow as monolayers/cell suspension cultures that can be passed, without the use of enzymes to expand the cell strain (s) for use in manufacture of cell based wound healing devices and as a source of stem-like cells for undertaking the chemical epigenetic manipulation of somatic cells with small molecules [9].

## Methods

### Primary keratinocytes

Primary oral keratinocytes were established as described [10]. The biopsy material was from two sites: the roof of the mouth which is keratinized epithelium or from the cheek area which is non-keratinized. The material was a punch biopsy or a surgically removed full thickness piece. The tissue was soaked in Ca^++^ and Mg^++^ free PBS containing 25 μg/ml Gentamycin and 0.375 μg/ml Amphotericin B (Fungizone)(both from Gibco, Life Technologies, New York). The punch biopsy or full thickness epithelium was sliced into ¼ inch (6.35 mm) wide strips, to facilitate trypsinization. The tissue was overlaid with 0.25 gm% type 1X-S trypsin in Ca^++^ and Mg^++^ free PBS (Sigma, St. Louis). Trypsinization was overnight, at room temperature. This separates the dermis from the epidermis and allows the basal cells to be gently shaken from the tissue. The primary adult human epidermal keratinocyte cultures were prepared as described in Marcelo et al [11]. The tissue was provided by the University Of Michigan Dept. of Surgery, section of Plastic Surgery, from elective surgeries. The tissue was full thickness, often with some adipose tissue. The dermal component contained blood derived cells which were not completely removed with several changes of Ca^++^ and Mg^++^ free PBS with antibiotics. After slicing the tissue into ¼ inch wide strips the tissue was trypsinized overnight at room temperature. The trypsin was removed, complete medium plus 2% fetal calf or newborn calf serum (Gibco, Life technologies, New York) was added to the 100 mm Petri dishes containing the tissue. The tissue was split at the dermis/epidermis junction and the epidermal portion was easily removed with forceps, and shaken to release attached cells. The dermis was gently scraped to remove attached cells. The cell suspension contained basal cells and spinous cells, and blood cells (RBCs, lymphocytes etc.). The cell suspension was filtered using Millipore 150 or 240 micron mesh, centrifuged at 208.5 xg at room temperature, for 5–10 minutes. The soft pellet was re-suspended in 2% serum in complete medium plus antibiotics. Fifteen ml of cell suspension containing a total of 10–20 x10^6^ cells at 95% viability were plated per a T-75 flask. The medium was removed and replace with complete growth medium with no serum after 24–48 hrs. The plating efficiency was 5–10%. Cells were grown in Epilife® supplemented with defined growth factors (EDGS) and 0.06 mM Ca^2+^ (INVITROGEN Co., Carlsbad, CA, Life Technologies, New York) plus antibiotics. Urothelial cells were cultured from ureteral tissues as previously described [12] and grown in a low-calcium, serum-free growth medium (Keratinocyte-SFM (KGSF) supplemented with 0.2 ng/ml of epidermal growth factor, 30 μg/ml of bovine pituitary extract, and 1% penicillin-streptomycin, (INVITROGEN, Co, Carlsbad, CA, Life Technologies, New York); the cells were grown in Epilife® plus EDGS for ePUK studies. Cells were grown at 37°C, 5% CO_2_ gassing and atmospheric oxygen. Corning or Costar brand tissue culture flasks centrifuge tubes and other disposable wares were purchased from Fisher Scientific, Pittsburg, PA.

### ePUK culture

**Figure 1:**
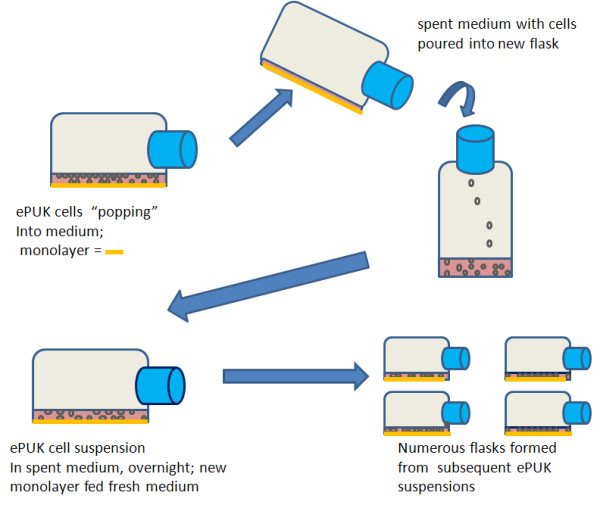
**ePUK protocol.** In the early passages there were 86,000 ±1,275 (SEM) cells/ml, with a medium number of 78,000 cells/ml, 79% viability, n=10, which is a cell production of 2.3 X10^6^ cell/T-75 flask, per day.

Primary epithelial cells isolated from adult human epidermis or oral mucosa at the first medium change or at minimum 30% confluence were fed complete Epilife Growth medium adding 35 ml/T-75 (15 ml/T-25 flask), every 24 hr. At confluence, the monolayers continued to proliferative, pushing keratinocytes into the overlying medium. The cells in suspension, named ePUKs (epithelial pop-Up keratinocytes) are poured into a new flask, and form new cultures, as depicted in Figure 1. The urothelial cells were obtained as P1 primary cultures. Addition of stock solution of glucose to bring spend medium to approximately 100 mg/100 ml was done, but did not consistently improve the passage of the ePUK cells (data not shown).

### Glucose assay

Spent and fresh medium was assayed for glucose using the o-Toluidine reaction [13]. The o-Toluidine was purchased from Aldrich-Sigma (St. Louis, MO) and the glacial acetic acid was obtained from Fisher, Pittsburg, PA.

### Cell number and size distribution

Epithelial cell suspensions isolated from tissue, ePUK cell suspensions, and trypsinized monolayer cells were counted and analyzed for size distribution using a Countess® Automated cell counter (INVITROGEN, Carlsbad, CA, Life Technologies, New York). The small cell population was 8–11 microns in diameter when cells were grown serum- and fatty acid-free in low calcium medium. Human red blood cells read 6–8 microns in diameter on this instrument and thus 8 microns was used as the cut-off for keratinocyte cell size analysis since blood cells are present in the initial cell suspension and in the first passage(s) as contaminating cells.

### Addition of fatty acids

Essential fatty acids 18:2, n-6 and 20:4, n-6 were attached to bovine serum albumin and added to the medium as described by us [14]. Studies show that 16:0 is necessary for successful incorporation of the fatty acids into cellular membranes, and for the phenotypic changes induced by the fatty acids to occur. Fatty acids were purchased from Nu-Check Prep, Inc., Elysian, Minnesota.

### Attachment of ePUK cells on microbeads and spheroid formation

Cytodex® microcarrier beads were C3275 (cytodex 3, 60–87 μm bead size, dextran beads) purchased from Sigma-Aldrich St. Louis, Mo. 1.0 g beads were soaked 3 hrs. in 100 ml PBS, Ca^++^ and Mg^++^ free, after which PBS was discarded, replaced with 50 fresh PBS and autoclaved for 15 min. Beads settled, PBS was suctioned off, and 30 ml of an ePUK P1Sn cell suspension was added; after 1 hr. at 37°C, the beads and ePUK cell suspension was moved to a T-25 flask and incubated. For this study, enough glucose was added to the spent medium/ePUK suspension to bring the glucose concentration to approximately 90–100 mg/100 ml. The culture was monitored by microscopy.

To search for spheroid formation epidermal ePUKs were grown in non-tissue culture surfaced vessels, and glass culture vessels to force growth in suspension. Visual scrutiny and cell size analysis by Countess® cell automated counter of the epidermis, oral mucosa and urothelial derived ePUK cells in suspension was done.

### EPUK growth on air/medium interface device

Growth of oral mucosa and epithelial keratinocytes on a dermal equivalent, AlloDerm®, was done as reported by us [10]. EVPOME is a composite human equivalent consisting of a dermal component composed of a human cadaver dermis, AlloDerm®, (LifeCell, Branchburg, NJ, USA) that is seeded with autogenous human oral or epidermal derived keratinocytes to form an overlying stratified parakeratinized epithelial layer. The epithelial keratinocytes were grown as ePUKs. The AlloDerm® was pre-soaked in 19 μg human type IV collagen (Becton Dickinson Labware, Bedford, MA, USA) for three hours prior to seeding to enhance adherence of the seeded keratinocytes (1.25 × 10^5^ cells/cm^2^). The composites of keratinocytes and AlloDerm® were then cultured, submerged, for 4 days to form a continuous epithelial monolayer. Then the concentration of calcium in the culture medium was raised to 1.2 mM to enhance keratinocyte differentiation to encourage stratification of the epithelial monolayer. When the equivalents are raised to an air-liquid interface they are cultured for an additional seven days to enhance differentiation and stratification resulting in the formation of a parakeratinized layer.

### Immunohistochemistry

ePUK cell suspensions were cytospun at 750 rpm for 5 minutes onto slides using a ThermoShandon Cytopsin 4 (ThermoShandon, Pittsburg, PA; Shandon Cytoslide and ThermoShandon single cytology Funnel, from Fisher Scientific, Pittsburg, PA). The slides were fixed in 95% ethanol for 24 hr and pre-treated for antigen retrieval for 10 minutes in pH 6.0 citrate.

Primary monoclonal antibodies were used and counter staining was done with Hematoxylin and processed for visualization using a Nikon E-880 microscope.

Antibody CD71 was from Novus Biologicals, Littleton, Co, (NB100-92243); Antibody Cytokeratin 14 (5), Novus Biological, NC110-59922; Antibody Cytokeratin 10, Novus Biological, NBPI-22537; Antibody Involucrin, Sigma-Aldrich, St Louis, MO, Cat # 19018; Antibody ITGA6 (α_6_β_4_ integrin), Sigma-Aldrich, Cat # WH0003655m1; Antibody Ki67, DAKO, Carpinteria, CA, Cat# M 7240; Antibody LORICIN, Sigma-Aldrich, Cat # AV 41738; Antibody p63, NeoMarker/ LabVision, Fremont, CA, Cat# MS 1081, Antibody filaggrin, Abcam, Cambridge, MA, mouse monoclonal, Cat # ab3137. EVPOMEs were processed as reported by us [10].

### Statistical analysis

Statistical analysis was undertaken using 2-way Anova and unpaired T-test, two tailed (GraphPad Prism 5).

### Procurement of human epithelial tissue

Keratinized and non-keratinized oral mucosa was collected from patients undergoing minor oral surgery procedures. The Institutional Review Board at the University of Michigan Medical School approved use of the mucosa, and patients provided informed consent for research use. The urothelial cells were prepared by Dr. Monica Liebert and were gifted to CLM. The oral mucosal tissue was collected and processed under Institutional Board approval at the University of Michigan Medical School and the patients provided informed consent. The epidermal tissue was discarded material from adult patients undergoing breast reduction procedures. In some instances, abdominoplasty skin was also used. No Institutional board approval was required and the patients provided consent via general University of Michigan consent. The study adhered to the Declaration of Helsinki Guidelines.

## Results

### Epithelial pop-up keratinocyte (ePUK) technique

Epithelial keratinocytes were prepared as reported [10-12]. For this technique, primary (P0) cultures were fed with approximately 30–35 ml of Epilife® low calcium medium plus EDGS supplement in contrast to the standard volume of 15 ml/T-75 flask.

All the epithelial cell types studied, i.e. epidermal, oral mucosa (both keratinized and non-keratinized) and urothelial grew in ePUK culture showed similar patterns of growth and expansion and cell size, had similar rates of glucose utilization, and exhibited epithelial phenotypic markers specific to their cell types. For brevity, data sets for one epithelial cell type are presented for each study. Also, epidermal derived cells in suspension and cultures were used in studies that needed large numbers of tissue-derived suspensions and cultures, since there was an abundance of this tissue available to us. Growth in a cell based device (EVPOME) is presented for epidermal and oral mucosal cells and attachment on microbeads was only tested using epidermal derived cells since our interest on this type of growth was specific to this cell type.

**Figure 2:**
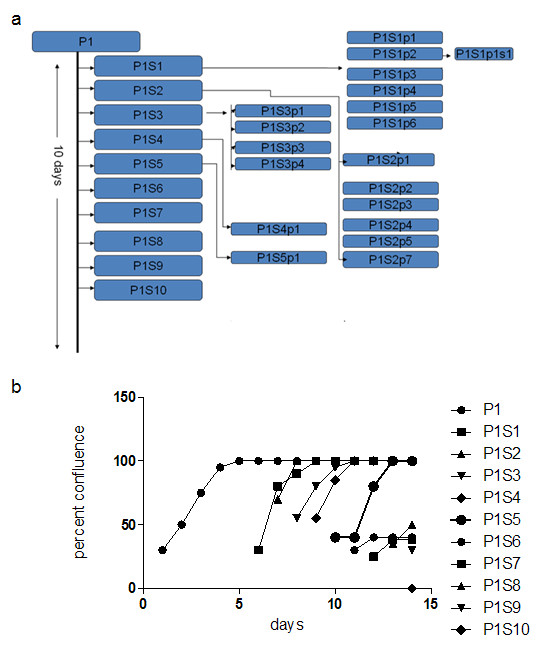
**Growth and expansion of ePUK culture.** 2**a**: Ten T-75 flasks were produced in 10 days, one per day from one P1 flask. The P1Sn flasks became confluent, producing P1Snpn flasks, continuing to form additional generations of ePUKs for several months. 2**b**: This Figure presents the plating efficiency of the passages from Figure 2a. Passages P1S1 to P1S5, showed good plating efficiency reaching confluence after 3–4 days, while passages P1S6 to P1S10 demonstrate the decline of the original P1 flask and the vigor and viability of the ePUKs at the end of the 2 week period. This decline was always seen, as each generation of the cell line reach confluence, produced ePUKs and then became an “exhausted” monolayer. The ePUKs were established from urothelium.

The cultures quickly reached 100% confluence, did not contact inhibit (i.e. stopped proliferation and entered differentiation state for epithelial cells) but continued to proliferate, “popping” cells up into the overlying medium. Passage of the spent medium, after an 24 hr. growth period, containing the cell suspension into another T-75 flask produced a new monolayer having an initial 30-50% confluence, which quickly grew to create a new 100% confluent monolayer that also generated “pop-off” cells. Figure 1 is the passage protocol for the ePUK culture. Figure 2a &2b describe the growth characteristics of the ePUK passages.

Over 10 days a passage 1 flask (P1) produced 1 new flask each day (P1S1 to10,) and these newly seeded flasks reached confluence, each producing ePUKs that when seeded into new flasks formed additional monolayers which continued to grow. As shown in Figure 2a, one flask produced 30 flasks over a period of 10 days. Figure 2b indicates that the ePUK monolayers had approximately 25-30% confluence 16 hrs. after plating, and grew to 100% confluence over 2–3 days’ time. Note that the growth potential of the ePUK monolayers lessened in the latter passages P1S6 to 10, reaching confluence over a 7–8 days (data not depicted).

**Figure 3:**
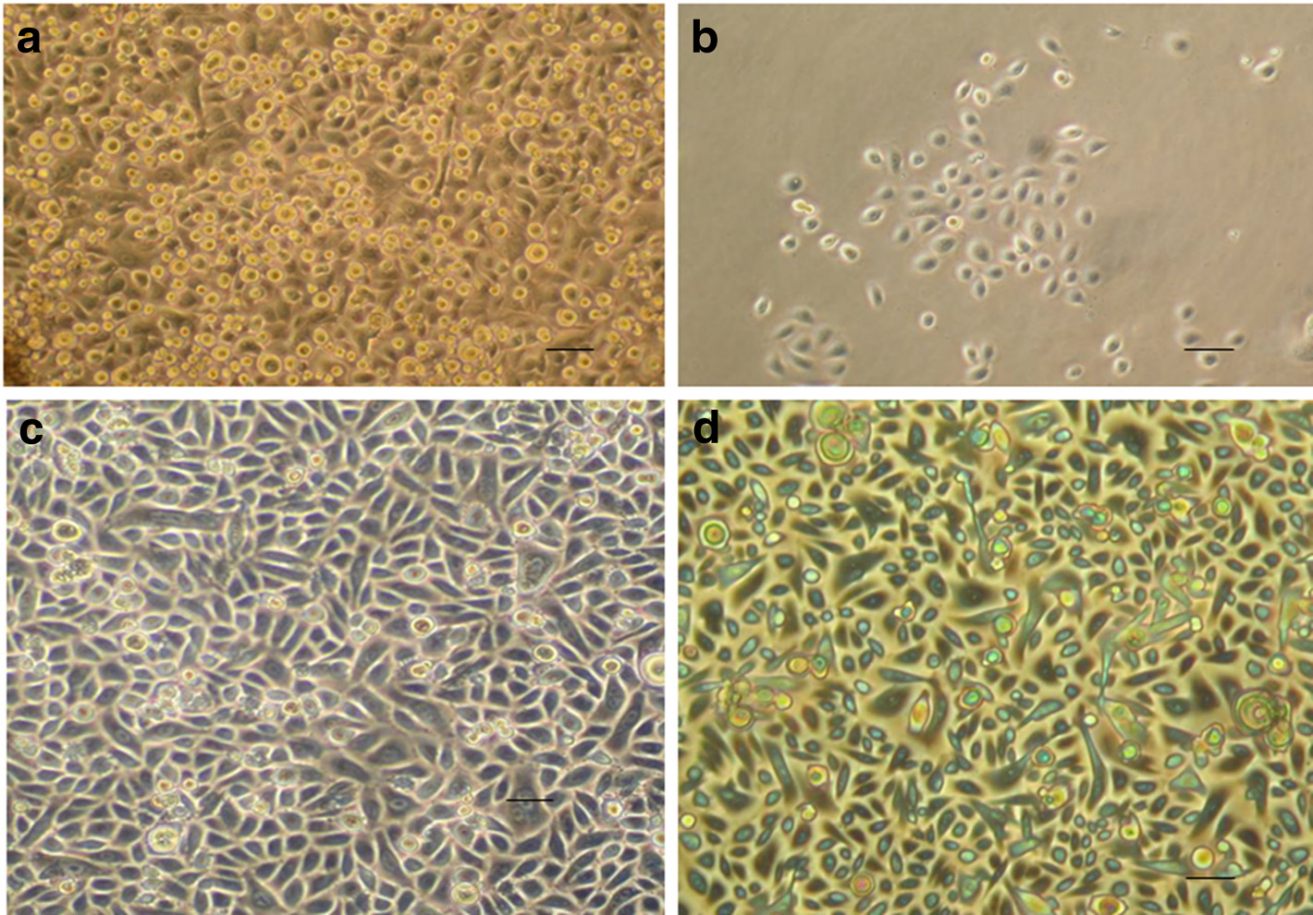
**Micrographs of ePUK cultures.** 3**a**: A complete monolayer is shown with ePUK cells in overlying medium. 3**b**: Adhering keratinocytes cells attached from cells suspended in spent medium when suspension was passed into a new flask. This micrograph was a taken approx. 16 hours after suspension was poured into new flask. 3**c**: A new monolayer formed after 2–3 days. 3**d**: A monolayer, after a number of generations (PnSnpnsn) of ePUK culturing demonstrated the very small population of epithelial cells that dominate an optimum monolayer grown using ePUK technology. Bar=50 microns for a,b,c,d.

Figure 3 a-d are photomicrographs of the monolayers derived from the ePUK technique. Figure 3a shows a monolayer of epidermis-derived cells with floating ePUK cells. In Figure 3b newly passed cells can be seen 16 hrs. after plating. These cells will form a new monolayer after 2–3 days (Figure 3c). Figure 3d is a 3^rd^ generation (PnSnpn as in Figure 2a) culture demonstrating a small population of keratinocytes which can dominate the ePUK cultures.

Any break in the ePUK protocol resulted in reduced cell number and viability in the ePUK suspension, lower plating efficiency, increased time to confluence, and increased cell size of the cell in the monolayer as visualized by microscopy. Testing epithelial cells growing in T-75 flasks (15 ml medium volume) showed that neither 15 nor 20 ml/flask daily medium change produced ePUK suspensions, while 30–40 ml of medium induced significant ePUK production. If the ePUK producing cultures were not fed after 24 hours, i.e., 48 hr., the cells in the monolayer that produce the ePUKs lost the rounded, small cell appearance began to flatten, the cytoplasm becoming more granular and “gray”, and the ePUKs in suspension did not plate when passaged.

### Characterization of ePUK cells

#### Cell size

**Figure 4:**
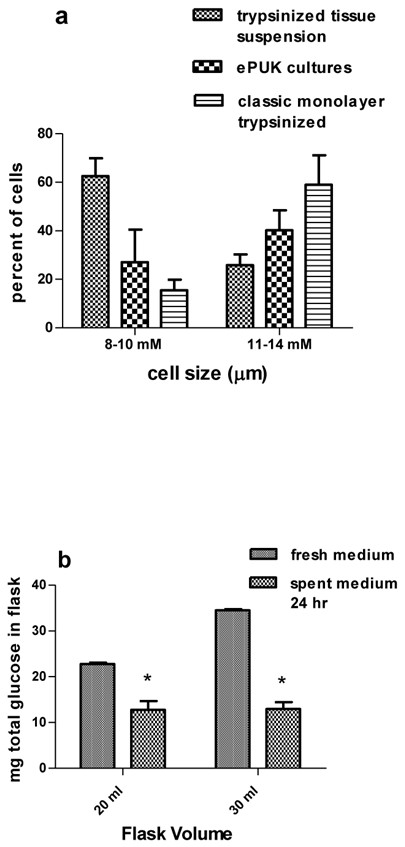
**Size and glucose utilization by ePUK cells.** 4**a**: ePUKs P1Sn and P1SNpn were studied. The cell suspensions produced from trypsinized tissue and size filtration of the resulting cell suspension consisted of 62.5% ±3.6 (SEM) small diameter cells (8–10 micron). The classically grown and passed P1,P2 cells contained 15.5% ± 2.2 small diameter cells while the ePUK cultures consisted of 27.1% ±4.3 micron cells. ePUK values n = 9, cell suspension n = 4 and classical monolayer n = 4 were compared using 2 way ANOVA and all means were significantly different at P ≤ 0.05. 4**b**: 20 ml of fresh medium contained 23 ± 0.3 mg glucose (approx. 114 mg/100 ml X 20 ml, n = 3) and 12.8 ±0.8 mg glucose after 24 hr. (n = 5). 30 ml of medium contained 34.4 ± 0.4 mg glucose (approx. 114 mg/100 ml X30 ml, n = 3) and 13.0 ± 0.7 mg of glucose after 24 hr. (n = 6), *P < 0.05 2 way ANOVA.

The ePUK culture technique maintains a larger portion of small diameter cells in culture as seen by microscopy**.** As presented in Figure 4a the presence of a smaller cell population in the ePUK cultures was verified using a Countess® instrument for cell counting and size distribution. The ePUK cells, generated from primary adult epidermis (breast) were P0S1P1 to P0S1P6 (defined in Figure 2a). The trypsinized tissue suspension was prepared from the skin used to generate the cells that formed the cultures, and the classically maintained monolayers were from parallel cultures grown using the traditional medium volume and feeding schedule (15 m//T-75 flask fed every 48 hrs.). The ePUK culture maintained a greater fraction of smaller cell population showing an approximate doubling of the percentage of small diameter cells that have increased prolific capacity, as reported by others [15]. ePUK values n = 9, cell suspension n = 4 and classical monolayer n = 4 and a field containing 200–300 cells was counted for each experiment, two chambers each. Each experiment (n) was a unique tissue sample.

#### Glucose utilization

Growth medium glucose level was used as an indicator of metabolic activity. The epidermis derived ePUKs utilized approximately 50% of medium glucose over a 24 hr. period. The T-flasks contained either 20 or 30 ml of medium and all the cultures were 90 + % confluent the time of analysis. In some cultures, the medium glucose could be so low that it did not measure in our assay which reads down to 30 mg/dl. The data is expressed as total amount of glucose/flask, indicating use of medium glucose by both the ePUK monolayer and cells in the overlying suspension. Expression of the data on a per cell basis was not done because studies to determine the relative contribution of the monolayer cells verses the ePUK cell suspension to glucose utilization was not examined, and the experimental protocol for this study required preservation of the monolayer component of the culture (the monolayer cells were not trypsinized and could not be counted).

This data emphasized the rapid consumption of glucose by the culture over 24 hrs. suggesting that low medium glucose levels could be limiting cell growth. Supplementing an ePUK culture at the 24 hr point with glucose to reach a 100 mg/dl fresh medium level and allowing the cells to continue in culture for another 24 hrs. resulted in ePUK suspensions that did not plate when passed (data not shown). This suggested that other medium components became exhausted or some cell product accumulated in the medium.

#### Phenotypic markers

**Figure 5:**
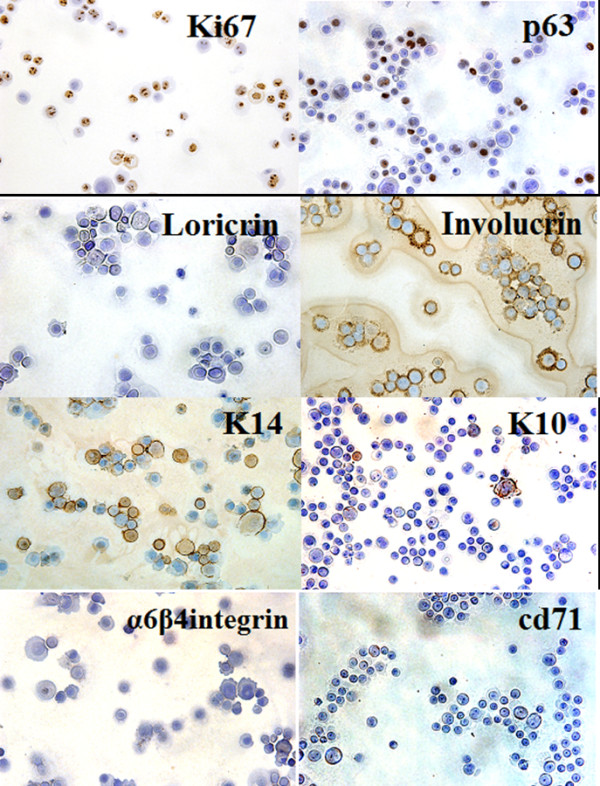
**Immunohistochemical staining of ePUK cells for epithelial cell markers.** Anti-Ki67, -p63, involucrin, and -cd71 antibodies are testing epidermal derived ePUKs; anti-k14, -k10, and -α6β-4 integrin antibodies are testing keratinized mucosa derived ePuks and anti-loricrin antibody is testing non-keratinized mucosal ePUKs. Positive staining is brown, and counter stain is blue. The ePUK cells are identified as cycling (+ for Ki67) basal cells (+ for p63 and K14). Non-keratinized oral mucosal is negative for Loricrin, and epidermal ePUKs showed staining for involucrin. Bar = 50 micron.

Using Cytospun® epidermal and oral mucosal ePUK cells and Immunohistochemistry it was demonstrated that ePUKs maintain their epithelial lineage over ePUK passage (Table 1, Figure 5). The epidermis derived ePUK cells in early passage (P2S2p2 and P2S2p2sn) are 66 ± 8% cycling cells (Ki67 marker), are mostly basal cells (p63, K14 markers) [16-18], and 4 ± 2% express the supra-basal cell marker loricrin [19]. The oral mucosa derived ePUKs demonstrated similar staining patterns, except for loricrin which is not a non-keratinized oral mucosa tissue marker [20]. The α6β4 integin^bri^/CD71^dim^ phenotype is reported to indicate epithelial stem cells [21], but this marker pattern was not significantly present in either the epidermal or oral mucosa derived ePUK cultures. Both epidermal and oral mucosa ePUks showed staining for involucrin which was very faint and diffuse, but was present on a large number of cells. This is contrary to published results, which indicate that involucrin does not stain keratinocytes in culture or the basal cells of the epidermis [22], although Watt et al have reported some involucrin staining on dividing cells grown from epidermis [23].

**Table 1:** Immunohistochemical characterization of epidermal and oral mucosal derived ePUKs

**MARKER**	**Epidermal**^**1**^** ePUKs**	**Oral mucosal**^**2**^** ePUKs**	**FUNCTION**
Ki 67^3^	66 ± 8%	25 – 50%	cell cycle related nuclear protein expressed by proliferating cells in all phases of the active cell cycle (G1, S, G2 and M phase) but absent in resting (G0) cells
P63^4^	64 ± 0%	20 – 75%	expressed in the epithelial basal cells of different organs and have been considered as possible markers of stem cells/reserve cells
K14^5^	75 ± 8	50 – 100%	expressed in basal cells of epithelium
K10^5^	16 ± 6%	20 – 30%	seen in all suprabasal cell layers including stratum corneum
loricrin	4 ± 2%^6^	0%^7^	major protein component of the cornified cell envelope found in terminally differentiated epidermal cells but not oral non-keratinized mucosal cells
involucrin^8^	88 ± 6 but very light and diffuse	100% light and diffuse	Found in terminally differentiated keratinocytes
α6β4integrin^9^	trace	Trace	α6 β4 complex receptor for laminin and It is expressed on epithelial basal cells
CD71^7^	trace	0%	α6 β4integrin^bright^/CD71^dim^ is reported to be a marker of epithelial stem cells

^1^Mean ± SEM, N = 4.

^2^N = 2, range.

^3^Knagg et al (1994) [16].

^4^Pellegrini et al (2001) [17].

^5^Lane et al (2004) [18].

^6^Mehrel et al (1990) [19].

^7^Lee et al (2000) [20].

^8^Watt et al (1981) [22].

^9^Webb et al (2004) [21].

### Induction of ePUK cell monolayer aging

**Figure 6:**
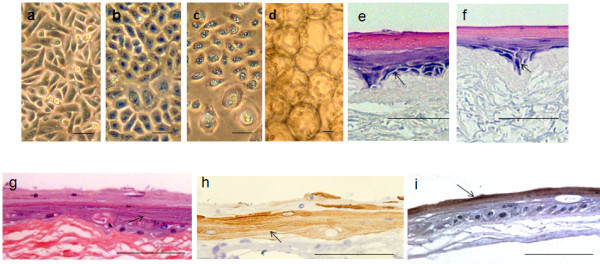
**Effect of essential fatty acids on ePUKs morphology, ePUKs on microbeads, and growth on EVPOME.** 6**a**-**c**: Essential fatty acids and palmitic acid (16:0) were prepared and added to the medium: 5 μM 16:0, and 10 μM 18:2 (n-6) and 20:4 (n-6) bound to bovine serum albumin as carrier. a: control T = 0, b = 24 hr. and c = 48 hr. culture. Bar = 50 microns. 6**d**: The ePUK cells can be transferred to microcarrier beads. Bar = 50 microns. 6**e** and **f**: Mucosal cells grown from the retromoral pad were placed on Alloderm®, air lifted for 11 days and processed. Stain was Hematoxylin/Eosin, arrows denote basal layer and the stratified differentiated layers stain pink. e = ePUK cells and f = monolayer from which the cells were derived. The ePUKS were at P1S1 as defined in Figure 2a.The is Bar = 100 microns. 6 **g** and **h**: adult human epidermis derived ePUKs, P1S1p3 (Figure 2a), formed a stratified multilayered structure on Alloderm®, day 11; stained with H&E (Figure 6g). The structure stained for k10 keratins (Figure 6h), specific for epithelial differentiation in the uppermost layers (arrow). Bar is 100 microns. 6**i**: adult human epidermis derived ePUKs formed an EVPOME using P1S1p3 cells as defined in Figure 2a. Figure 6 i = an EVPOME stained with anti-filaggrin antibody showing production of this epidermal differentiation-specific protein by the upper most layer formed by ePUK derived cells (arrow). Bar = 100 microns.

As previously reported by us, keratinocytes grown in low calcium and serum-free conditions cells are essential fatty acid deficient. When essential fatty acids are added to the medium thus restoring these fats to membrane phospholipids and triglycerides, the cells proliferate much slower, the cells show signs of differentiation and the cultures age and die [14]. The monolayers created from ePUK cells acted in a similar fashion when essential fatty acids (10 μM 18:2, n-6 (linoleic acid) and 20:4, n-6 (arachidonic acid) in the presence of 5 μM 16:0 (palmitic acid), bound to bovine serum albumin as carrier were added to the medium. Matched ePUK T-75 culture flasks were grown in 15 ml, and 35 ml of Epilife® Medium with and without added essential fatty acids, for 24, 48 hrs.; only the flasks grown in 35 ml Epilife® produced ePUK cell suspensions. The flask fed 15 ml, every 24 hrs. contained a monolayer of larger cells, with signs of multilayering, and produced no ePUKs. The cells fed with 35 ml of medium plus essential fatty acids were larger, differentiating and showed signs of senescence, and no ePUKs were produced (Figure 6a-c). The cells also were induced to slow growth and to differentiate when 10% newborn calf serum was added to the growth medium or when medium calcium levels were restored to physiologic levels (1.2 mM)(data not shown).

### ePUK cell attachment on carrier beads

The ePUK cells are adaptable, and can easily attach to on microcarrier beads, a process useful for the production of cell-based devices [24-26] (Figure 6 d). This observation is important because a device using epidermal keratinocytes attached to carrier beads has been developed for clinical use and the ability of ePUK grown cells to be transferred to carried beads will optimize the production of this device, i.e., the cells are grown, passed and expanded without trypsin or other enzyme.

### ePUK cell growth on air/interface device (EVPOME: *Ex vivo* produced oral mucosa equivalent)

As seen in Figure 6 e-j, ePUK cells form a multilayered device that can be used for transplant as do keratinocytes trypsinized from monolayer culture. Figures 6 e and f were constructed from early ePUK culture of oral mucosal tissue. Figure e structure was formed from ePUK cells in suspension and Figure f from the monolayer from which the ePUKS were produced, after the cells were released by trypsinization from the monolayer. The arrow points to the basal layer, stained blue with H&E growing on the Alloderm® dermal layer. The red/pink stain marks differentiated cell layers. Figures 6g and h are micrographs of EVPOMEs formed from later passage ePUKS from a primary adult epidermis cell culture. Figure 6g is H&E stain of the structure shown in Figure 6h which stained positive for k10 keratin, a marker of epithelial differentiation (arrow) [18]. Figure 6g showed signs of granular layer formation (arrow). Figure 6i demonstrates the formation of filaggrin protein by the cells forming the EVPOME (arrow).

### Spheroid growth in ePUK suspensions

No evidence of cells growing as spheroids was seen in any ePUKs derived from adult epidermal tissue, from oral mucosa or in several cell strains derived from adult human urothelial tissue (data not shown) [27].

### ePUK fragility

It was observed when developing this technology that ePUK cells were fragile to physical handling and also required spent medium for passage. To demonstrate this phenomenon, a second or third generation ePUK cell suspension (PnSnpn Figure 2a) was separated into 3 fractions: 1) spent medium, cells not centrifuged, poured into new flask (control); 2) spent medium, cells were centrifuged and re-suspended in spent medium and then plated and 3) cells were centrifuged and re-suspended in fresh medium and then plated. Centrifugation was 1000 rpm (208.5Xg) at room temperature for 5–8 minutes (Sorvall, RT6000B, Dupont, Delaware). After 5 days in culture, the control cells were at 100% confluence, the centrifuged/spent medium cells had 70% confluence, and eventually grew to 100% confluence as the control, while the centrifuged/fresh medium ePUK derived monolayers were at 25% confluence after 5 days, and never formed a complete monolayer. This fragility was documented in two strains derived from epidermis and one from urothelial tissue and always seen in the many oral mucosa and epidermis strains that were grown using ePUK technology. Additionally, trypsinization of the newly plated ePUK cultures caused destruction of the cells by the enzyme action and pipetting of the suspension, so that we could not rigorously determine the plating efficiency of the ePUK suspension. This is very unusual for keratinocytes since in our hands they are quite hardy once established in culture and grown using routine culturing protocols. We have no explanation for this observation, but report ePUK cell fragility as an important technical point.

## Discussion

Epithelial cells, including those isolated from the epidermis consist of a family of cells: stem cells, progenitor or transitional cells and differentiated cells. Stem cells can be defined as cells that have the potential to divide and to produce a replica cell as well as differentiated progeny and are thought to last the lifetime of the organism [15]. In the interfollicular epidermis (non-hair follicle) and in the oral mucosa and other epithelia, these cells are reported to have specific expression patterns of several cell surface markers [21], and *in vitro* are reported to be “small’ in size (15–20 microns in diameter when attached to the growth surface) [15]. Progenitor or transitional cells are dividing cells committed to differentiation, are larger (30–40 microns) and the differentiated cells are greater than 40 microns and have a distinctive “differentiated” appearance in culture.

In routine tissue culture of epithelia cells, it is thought that some stem cells are in the initial culture, along with progenitor or transitional cells but that the stem cells are “lost” during culture growth and passage, with progenitor cells forming the primary cultures, with limited life-span.

The routine culture of primary keratinocytes from skin and other epithelia involves changing the spent medium over the cell monolayer every second day with standard amount of medium (T-25 flask uses 5 ml of medium; a T-75 flask holds 15 ml of medium, etc.). At about 70% confluence, the monolayer is passed or split, using trypsin or dispase and if treated with care, a primary culture can be passed 7–12 times. In this type of “routine” keratinocyte culture, monolayer confluence induces differentiation and eventual cell death if the cells are not passed soon after reaching 100%.

As presented in this report, a technique has been developed that produced epithelial cell strains with a high percentage of small diameter cells. The cells have proliferative potential and grow in a coordinated monolayer/suspension. The technology has a number of unusual manipulations: the cells were fed once a day, with 2-3X the amount of medium, which is serum and fatty acid free and contains low calcium levels. The cells maintained active cell proliferation at 100% confluence so that the progeny cells were pushed or popped into the overlying medium. The spent medium cell suspension, containing about 80,000 cells/ml, at about 80% viability, was then poured into a new flask (Figure 1), where a majority of the cells then attached, forming a new monolayer. The new flask became confluent, the monolayer producing ePUK cells, which formed a new monolayer upon transfer to another flask, expanding the cell strain over 1–2 months’ time. The original flask stopped producing ePUKs, and the monolayer consisted of large, aged cells and “died” after 10–12 days.

The ePUKs were more fragile than their traditionally cultured counterparts, most notably being sensitive to centrifugation. It was thought that the cells might grow as spheroid cells, [27], since epithelial cells with enhanced growth potential can grow as bundles of cells in suspension, but no evidence of this type of cells growth was seen.

The ePUK producing monolayers appeared to have a high nutrient requirement, as indexed by high glucose utilization. It is possible that the rapid growth of the cells, and growth of the ePUKs in suspension after monolayer confluence, was simply because of frequent feeding of the primary epithelial cells with larger than routine amounts of medium. Beside the use of larger medium volumes, feeding the cells every day was also essential to the technology; failure to do either of these protocol requirements stopped the growth of the cells as ePUK cultures. Thus, the ePUK culture technique may, in part be providing the nutrients necessary for growth. This concept would suggest that constant feeding of the cells, as in a continuous feed bioreactor, may allow the cells to expand for a limitless number of passages allowing creation of master cell banks for use in cell based therapies.

The importance of nutrient levels in ePUK proliferation implicates cell signaling pathways such as mTOR as control points in this type of *in vitro* epithelial cell growth [28]. This pathway is also implicated by recent studies by us demonstrating that rapamycin, an inhibitor of mTOR(C1) allows for greatly expanded growth of oral mucosal epithelial cells in monolayer cultures [29].

The data indicating that ePUK growth of keratinocytes maintains a smaller diameter cell fraction suggested that an early progenitor or stem-like cell was supported by this culture method since early progenitor/stem-like epithelial cells are theorized to be small diameter cells. The observation that ePUKs did not express the α6β4^bri^T/cd71^dim^ phenotype reported to indicate true epithelial stem cells [21], suggested that ePUKs were possibly early progenitor cells maintained by the serum and fatty acid-free low calcium medium that stopped differentiation and a frequent feeding schedule that supplied nutrients for cell growth. Alternately these cells could be “stem” like cells that are not expressing α6β4-integrin since they are in suspension and have no immediate need to express this receptor for laminin [22].

Moreover, in a recent publication Chaffer et al [30] report the spontaneous growth of a cell type from normal and neoplastic nonstem cells that can convert to a stem-like state. These cells are basal-like human mammary epithelial cells that came *de novo* from differentiated cells in culture, i.e. transdifferentiation, and float on top of existing cell monolayers. This conversion was observed in normal epithelial breast cells, the cells floated atop of the cell monolayer and it was stated that their presence was a rare occurrence. This report is intriguing because the cells are similar to ePUKs, which in our hands are routinely induced using simple technology, and are produced in large numbers (80,000/ml/day in flasks with 30–40 ml). It appears that ePUKs may be a stem-like cell that can be easily induced from adult epithelial tissues that do not express the α_6_β_4_^bri^/cd71^dim^ phenotype.

The *in vitro* phenotype of ePUK cells could be maintained because of the 1) extra nutrients provided to the cells by frequent feeding of 2-3X volumes of medium, and/or 2) some product made by the cells, as evidenced by the requirement for spent medium for successful passage of ePUK suspensions to form new monolayers, and/or 3) by the fact that the cells are never trypsinized or scraped for passage, so that the cell surface is never perturbed.

The cells in this report were primary adult cells in culture that were never passed using enzyme digestion and never cyropreserved. Application of this technology to adult human keratinocytes initially grown using traditional protocols, and passed with trypsin and cryopreserved (for as long as 2–3 yrs.) also yielded ePUK cells; These ePUKs grew with a shorter ePUK production period, and less ePUK expansion (data not shown). Neonatal foreskin keratinocytes were not used in these studies.

iPS cells have been induced from human foreskin keratinocytes using specific transcription factors that were introduced using retroviral vectors indicating that the keratinocyte cell type can be reprogrammed [4,31]. The large percentage of small diameter cells growing as ePUKs, in suspension, and lack of ePUK monolayer stratification and differentiation suggests that ePUK keratinocytes are more “plastic” in phenotype and that they may be a useful stem-cell like type for epigenetic manipulation with small molecules, as already been reported by other investigators using mouse keratinocyte cultures [9].

## Conclusions

We have shown in this work that human epithelia keratinocytes in primary culture can be induced by tissue culture manipulation to produce, without the use of enzymes for passaging, large numbers of small cells in a combined suspension/monolayer culture. These small cells maintain their epithelial characteristics and will revert to epithelial morphology when grown at the air-liquid interface on a skin equivalent model. For practical applications, the ePUK technology can be used to produce devices for wound healing and tissue engineering/regenerative medicine possibly in higher numbers and faster than with other culture techniques. The ePUK keratinocyte phenotype resulted from absence of serum-derived factors and manipulation of the calcium, essential fatty acid and metabolic nutritional status of the primary cultures. Thus keratinocytes in ePUK culture provide an additional controlled human primary cell system for investigation of the mechanisms regulating epithelia cell growth and differentiation. Additional studies are being conducted with this system because it provides an excellent possibility for the long-term maintenance of fresh, basal-like, non-differentiated, proliferative epithelial populations.

## Abbreviations

ePUK, epithelial Pop Up Keratinocytes; ESCs, embryonic derived stem cells; iPSCs, induced pluripotent stem cells; PnSnpn, Passage (0,1,2) subculture (1,2,3) passage 1,2,3) i.e., P1S1p1 is the first passage of the first subculture of the first Passage of the original plating of cells forming a culture (P0).; EVPOME, Ex vivo produced oral mucosa equivalent.

## Competing interests

The authors declare that they have no competing interests or conflicts.

## Authors’ contributions

CLM made the initial observation, conceived of the study, designed and carried out the cell growth and size studies, performed the glucose analysis and statistically analysis, interpreted the data and wrote the manuscript. AA assisted CLM in the cell growth and glucose analysis, AP carried out the immunohistochemical studies and helped in the final draft of the manuscript. SEF assisted in the interpretation of the data, and in the final draft of the manuscript. All authors read and approved the final manuscript.

## Pre-publication history

The pre-publication history for this paper can be accessed here:

http://www.biomedcentral.com/1471-5945/12/8/prepub
